# Optimization of Inhalation Technique Knowledge in the Pharmacies of Matosinhos Municipality, Portugal: An Intervention Project

**DOI:** 10.7759/cureus.50655

**Published:** 2023-12-17

**Authors:** Diana Rodrigues Pacheco, Cecília Vieira, Inês Freitas, Joaquim Santos, João Salgado, Patrícia Lopes

**Affiliations:** 1 Family Medicine, Unidade de Saúde Familiar Horizonte - Unidade Local de Saúde de Matosinhos, Matosinhos, PRT; 2 Family Medicine, Unidade de Saúde Familiar Leça - Unidade Local de Saúde de Matosinhos, Matosinhos, PRT; 3 Family Medicine, Unidade de Saúde Familiar Caravela - Unidade Local de Saúde de Matosinhos, Matosinhos, PRT; 4 Family Medicine, Unidade de Saúde Familiar Bom Porto - ACeS Porto Ocidental, Porto, PRT; 5 Family Medicine, Unidade de Saúde Familiar Covelo - ACeS Porto Oriental, Porto, PRT

**Keywords:** general practice, asthma, copd, pharmacies, inhalation device

## Abstract

Introduction: The therapeutic approach to the most common respiratory diseases, asthma and chronic obstructive pulmonary disease (COPD), involves the use of inhalation devices. Its use is essential, but incorrect use is frequent, and learning and reviewing the technique is necessary for the adequate management of these diseases.

Objective: This study aims to increase the knowledge of health professionals from pharmacies in the municipality of Matosinhos, Portugal, about the correct inhalation technique.

Methods: The project developed from May 2022 to June 2023 was based on a training session for pharmacy professionals that took place after professionals completed a questionnaire and after evaluating each participant's inhalation technique by the authors using a checklist. Both were reapplied three months after the first evaluation.

Results: Fifteen pharmacies in Matosinhos, Portugal, were invited, of which eight agreed to participate, with a total of 16 health professionals (30.8% of professionals in these pharmacies). We found that professionals questioned and taught users more times about the inhalation technique and that a greater number of professionals considered they knew how to correctly perform the inhalation technique in the second assessment. It was also possible to verify statistically significant differences (p<0.001) between the mean of correct steps in the inhalation technique between the first and second assessments. There was also an improvement in the qualitative analysis of errors in the inhalation technique. In the first assessment, the inhalation devices with the lowest percentage of correct steps were Forspiro® and Respimat®. In the second evaluation, the Diskus® and Ellipta® devices obtained 100% correct steps, with the K-haler® being the device with the lowest percentage.

Conclusions: This study confirms the effectiveness of continuous training for health professionals and the importance of multidisciplinary cooperation in teaching inhalation techniques. Other studies are needed to complement these results, particularly with representative samples of the general population, or assessing the impact of this intervention in patients with respiratory diseases.

## Introduction

Asthma and chronic obstructive pulmonary disease (COPD) are characterized by an obstruction of the airways, resulting in symptoms, such as dyspnea, cough, with or without sputum production, and wheezing. Both diseases are chronic, and during their course, exacerbations of varying severity may occur, necessitating healthcare interventions [1].

Their prevalence renders them the most common chronic respiratory diseases globally. It is estimated that asthma affects 262 million people worldwide, being the primary lung disease in children [2]. COPD, with a prevalence of approximately 300 million, ranks as the third leading cause of global mortality, accounting for 3.23 million deaths in 2019 [2]. In Portugal, the data align with other European countries: asthma affects 6.8% of the population, and COPD affects 14% of individuals over 40 years old [3,4].

The therapeutic approach to these diseases involves the use of inhalation devices. These devices may contain corticosteroids, short- or long-acting muscarinic antagonists, and/or short- or long-acting beta-agonists. For each of these drugs, there are different devices, each with distinct mechanisms of action, categorizable into three major groups: dry powder inhalers, soft mist inhalers, and pressurized metered-dose inhalers, with or without a spacer [5]. The multitude of combinations and the complexity of device usage contribute to a high rate of incorrect utilization [6]. It is believed that a significant proportion of hospitalizations could be prevented with improved inhalation technique [7], given the high efficacy of therapy in controlling and preventing exacerbations [8,9]. In a study conducted in the United States, it is estimated that of the annual $25 billion spent on inhalers, five to seven billion dollars are lost [10].

For the reasons stated, international guidelines recommend the involvement of all healthcare professionals, including pharmacists, in teaching inhalation technique [8], as a highly effective means [9] to ensure correct device usage by the patient, emphasizing the need for regular review.

In conclusion, considering that in Portugal, all users need to visit a pharmacy to purchase medication, the authors believe that pharmacists and pharmacy technicians can contribute to improving therapeutic adherence in asthma and COPD. In this context, an intervention project was developed with the primary goal of optimizing the knowledge of healthcare professionals in pharmacies in the municipality of Matosinhos regarding the correct inhalation technique, aiming to assess whether knowledge improves following the intervention. Secondary objectives were defined: to train at least 80% of professionals from the five pharmacies geographically closest to each family health unit and that at least 75% of these professionals correctly answer at least 80% of the checklist questions after the intervention.

This article was previously presented as an oral communication at the 9ª Jornadas do Grupo de Estudos de Doenças Respiratórias (GRESP) on October 19, 2023.

## Materials and methods

The intervention project was designed by the authors, who constituted the management and execution group for this study. The proposing entities were the three Family Health Units (FHUs) of the authors in the municipality of Matosinhos, Portugal. The timeframe spanned from May 2022 to June 2023. The target population comprised professionals from the five pharmacies geographically closest to the three mentioned FHUs. The project was presented and approved in a service meeting in all three FHUs, as well as approved by the Clinical and Health Council and the Ethics Committee for Health of the Local Health Unit of Matosinhos (LHU) (83/CES/JAS).

Professionals who agreed to participate in the study were invited to attend an in-person training session on inhalation techniques, led by a general practitioner specialist with relevant training in respiratory diseases. This session took place after professionals completed a brief questionnaire and after the authors assessed the inhalation technique of each participant using a checklist. The questionnaire included questions about the pharmacy professionals' practices regarding inhalation devices, such as the frequency of sales, inquiring about correct usage, and providing technique instructions. In case of a negative response, the reasons for not doing so were explored. The checklist allowed for the evaluation of knowledge about all steps of the inhalation technique for all assessed devices. One point was assigned for each step performed correctly, and the total score was expressed as the average of the correct steps.

In addition to evaluating the inhalation technique for each device, a qualitative analysis of the type of errors committed by device type was conducted. Thus, the inhalation technique of professionals making critical errors (failure in device preparation/activation and/or inhalation) was categorized as "poor." The inhalation technique with intermediate errors (especially forced expiration preceding inhalation and final breath-holding) was categorized as "in need of correction." The inhalation technique without the mentioned errors was considered "adequate."

The participants were invited to fill out the questionnaire again and were reevaluated with the checklist three months after the intervention to verify if the proposed objectives were achieved. To enhance project adherence, the authors visited the pharmacies for the second evaluation.

All participants provided informed, voluntary, and clear consent for study participation.

Results will be presented in absolute and relative frequencies and mean percentages. Statistical inference will be performed using the Wilcoxon test to assess whether the percentage of correct steps before and after the intervention differs. A p-value <0.05 was considered statistically significant. Statistical analysis will be conducted using SPSS Statistics for Windows, version 17.0 (released 2008, SPSS Inc., Chicago).

## Results

A total of 15 pharmacies were invited, corresponding to the five geographically closest to each FHU involved. Of these, eight agreed to participate, totaling 16 healthcare professionals, representing 30.8% of the total healthcare professionals from these eight pharmacies.

Table 1 presents the results of the questionnaire applied to pharmacy professionals regarding their daily practice in the two evaluations.

**Table 1:** Questionnaire on pharmacy professionals' practices. The data are represented as the total number of responses (n) and percentage (%).

	Number of responses	
Question	First evaluation (0 months) n (%)	Second evaluation (3 months) n (%)
1 - In the last month, did you sell inhalation devices?		
Yes	16 (100)	16 (100)
No	0 (0)	0 (0)
2 - In the last month, how many times did you inquire if the user knows how to perform the inhalation technique?		
0-25%	4 (25)	0 (0)
25-50%	2 (12.5)	3 (18.75)
50-75%	6 (37.5)	6 (37.5)
75-100%	4 (25)	7 (43.75)
3 - In the last month, what percentage of users did you review and teach the inhalation technique?		
0-25%	9 (56.25)	4 (25)
25-50%	4 (25)	7 (43.75)
50-75%	0 (0)	3 (18.75)
75-100%	3 (18.75)	2 (12.5)
4 - What were the main reasons that led you not to instruct the user on the inhalation technique?		
Forgetting	1 (6.25)	0 (0)
Lack of time	2 (12.5)	6 (37.5)
Lack of knowledge	3 (18.75)	0 (0)
Other	11 (68.75)	10 (62.5)
5 - Do you consider knowing how to perform the inhalation technique adequately?		
Yes	4 (25)	10 (62.5)
No	2 (12.5)	0 (0)
I have doubts	10 (62.5)	6 (37.5)

Through the analysis of Table 1, it is evident that, following the intervention, pharmacy professionals inquire and educate patients more frequently on inhalation techniques. In the second evaluation, there was no forgetfulness or lack of knowledge justifying the omission of patient education. However, "lack of time" emerged more frequently as a rationale for non-instruction. Various professionals indicated "Other" as a reason for not instructing the patient on inhalation technique, with "perceived patient already knew" and "chronic use" being cited as examples by professionals to justify this decision. It is noteworthy that a greater number of professionals considered themselves proficient in correctly executing the inhalation technique in the second evaluation, with none providing a negative response.

Table 2 presents the data from the assessment of the inhalation technique before and after intervention.

**Table 2:** Assessment of inhalation technique for all inhalation devices. The data are represented as mean and standard deviation (mean±SD) or total number (n), as specified.

	Before intervention	After intervention	p-value
Correct steps (mean±SD)	65.2 ± 20	90 ± 2.2	<0.001
Inhalation technique (n)			
Poor	95	12	
Needing correction	11	9	
Adequate	86	171	

There was an improvement in the average percentage of correct steps after the intervention, as observed in Table 2, and this difference was statistically significant (p<0.001). Regarding the assessment of the inhalation technique considering the type of errors committed by pharmacy professionals, we can observe that the training session led to a decrease in the "poor" and "in need of correction" categories, significantly increasing the "adequate" category.

**Figure 1:**
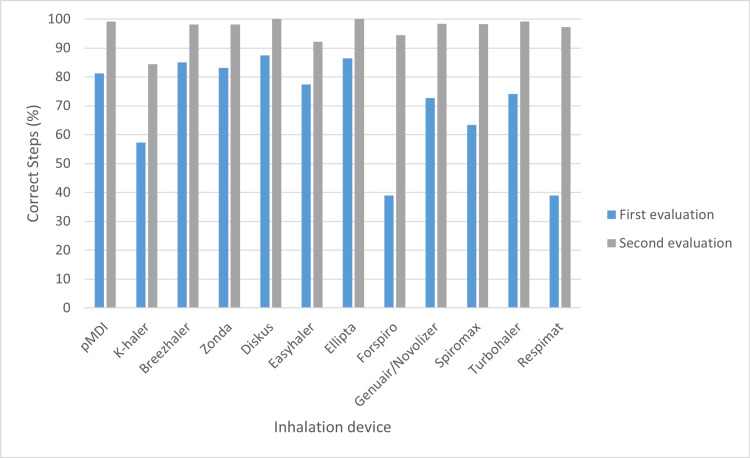
Percentage of correct steps per evaluated inhalation device.

In the initial assessment, the inhalation devices with the lowest percentage of correct steps were Forspiro® and Respimat® (Figure 1), both at 38.9%. In the second assessment, the Diskus® and Ellipta® devices showed 100% correct steps, while the K-haler® device had the lowest percentage (84.4%).

Regarding the most common error per device, it was observed that for pMDI®, K-haler®, and Easyhaler®, "shaking the device" was the most common error in both assessments. In six out of the 12 evaluated devices, the correct inhalation technique was the most commonly made error, and in three types of inhalers (Genuair/Novolizer®, Turbohaler®, and Respimat®), the most common error corresponded to the activation method of the device itself.

## Discussion

This study assessed the knowledge of healthcare professionals in performing inhalation techniques and the impacts of an educational intervention, confirming data on the significant impact of brief, low-cost, and time-efficient interventions on improving pharmacists' knowledge of inhalation techniques [11]. This information is particularly relevant when considering the potential impact on patients' lives. Gul et al. [12], in a 2023 article, not only demonstrated a statistically significant improvement in the inhalation technique of patients instructed by pharmacists but also an improvement in their quality of life. In another study, Basheti et al. quantified this improvement by assessing forced expiratory volume in 1 second (FEV1) and the use of rescue medication in rural areas of Jordan [13], finding that patients instructed by pharmacists had statistically superior results in spirometry and symptom assessment checklists. Several other articles have reported similar results, showing improvement in therapeutic adherence [14] and the use of rescue medication [15] for both asthma and COPD [16]. In our study, the fact that some devices were associated with a higher number of errors may be related to the prescription pattern in Portugal, where pharmacists are more familiar with certain techniques. In fact, in previous studies from other countries, there was no statistically significant difference between the types of devices [17,18].

To the best of our knowledge, our study represents a novelty compared to previous ones, as it evaluated all steps of the inhalation technique for 12 different devices, not just one type or individual technique per major mechanism of action (pressurized vs. dry powder vs. soft mist). In both assessment periods, the same researchers, and the fact that there was only one checklist adapted to each device, reduced the potential variability of results.

However, it is important to consider some limitations. First, the small sample size and the geographic limitation in pharmacy selection do not allow the results to be extrapolated to the general population. In addition, low adherence acts as a selection bias, making it difficult to infer conclusions from non-participants. The absence of a control group is also a study limitation.

On the other hand, the authors believe that the questionnaire directed at professionals may need to be reformulated, as the type of response may not accurately reflect their practices, such as not evaluating the average number of patients each professional attend to. The questionnaire responses should also include "0%," which could demonstrate the percentage of professionals who never question and never instruct patients on the inhalation technique.

## Conclusions

This study demonstrated the effectiveness of brief educational interventions in improving the knowledge of healthcare professionals in performing inhalation techniques, with the potential positive impact on patients' quality of life. In the future, it is crucial to expand the sample and diversify the regions studied and consider the inclusion of a control group, simultaneously assessing the impact on patients or long-term effects on disease control. Ultimately, empowering healthcare professionals to provide proper instruction on inhalation techniques plays a fundamental role in optimizing the control of respiratory diseases, benefiting both patients and the healthcare system.
